# Supplementary figures and images for: The regulation of a pigmentation gene in the formation of complex color patterns in Drosophila abdomens
Source: PLoS One. 2022 Dec 19;17(12):e0279061. doi: 10.1371/journal.pone.0279061 (PMC9762589; doi:10.1371/journal.pone.0279061)

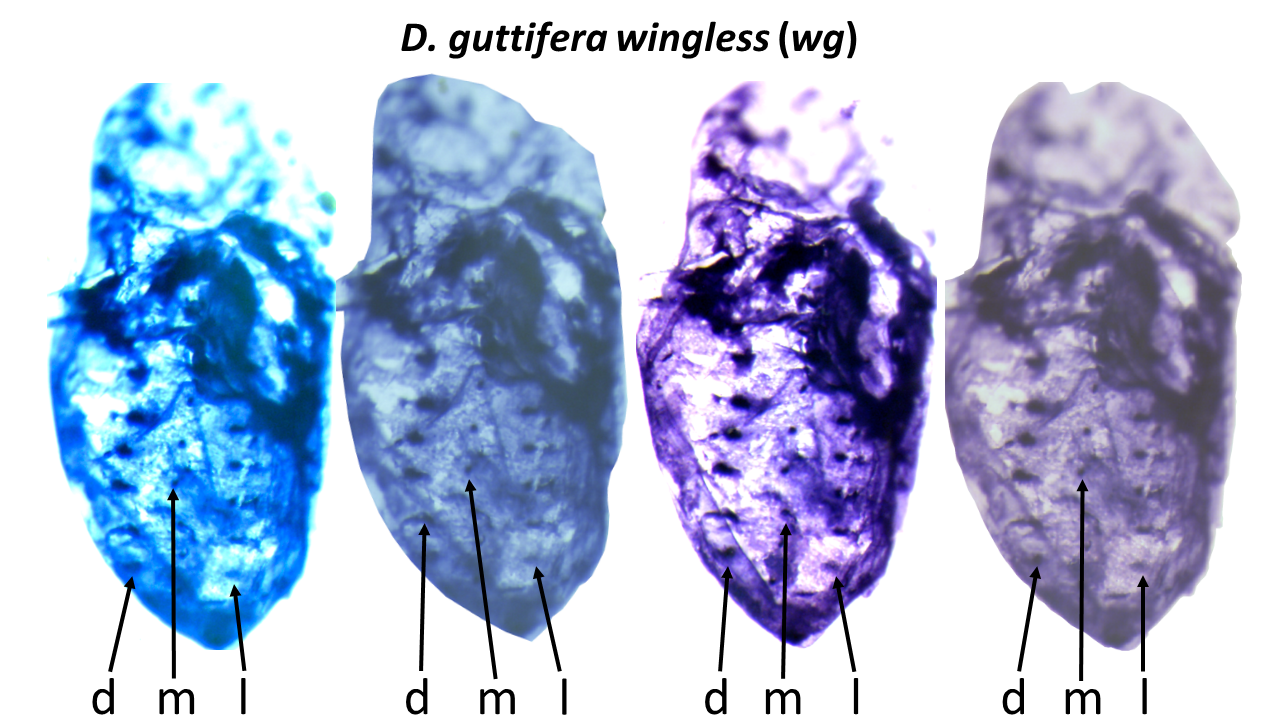

Supplement: S1 Fig — This is the same pupa as in Fig 2B, but with different image manipulations. d = dorsal, m = median, l = lateral row of spots. (TIF) [file pone.0279061.s001.tif]

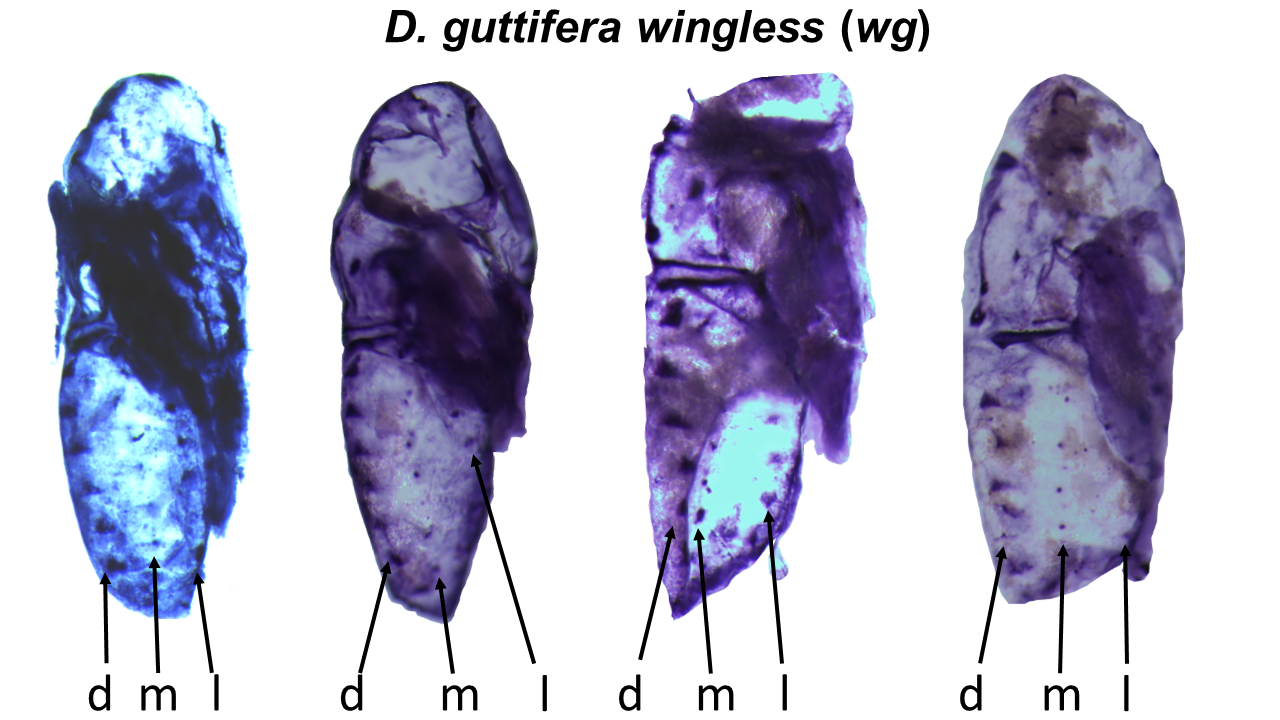

Supplement: S2 Fig — d = dorsal, m = median, l = lateral row of spots. (TIF) [file pone.0279061.s002.tif]

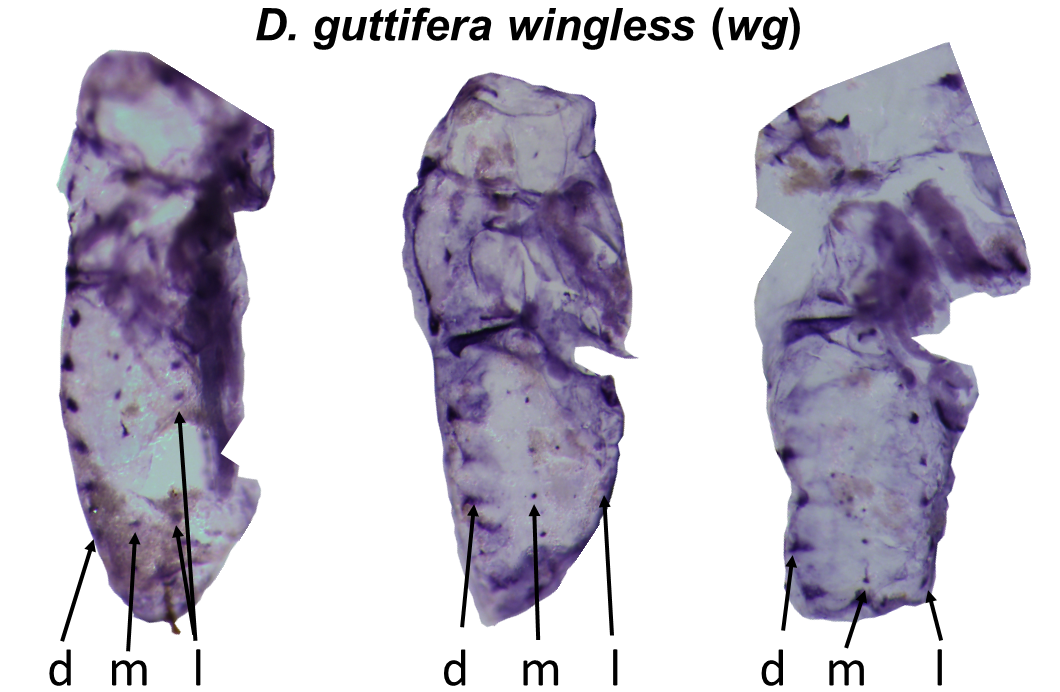

Supplement: S3 Fig — (TIF) [file pone.0279061.s003.tif]

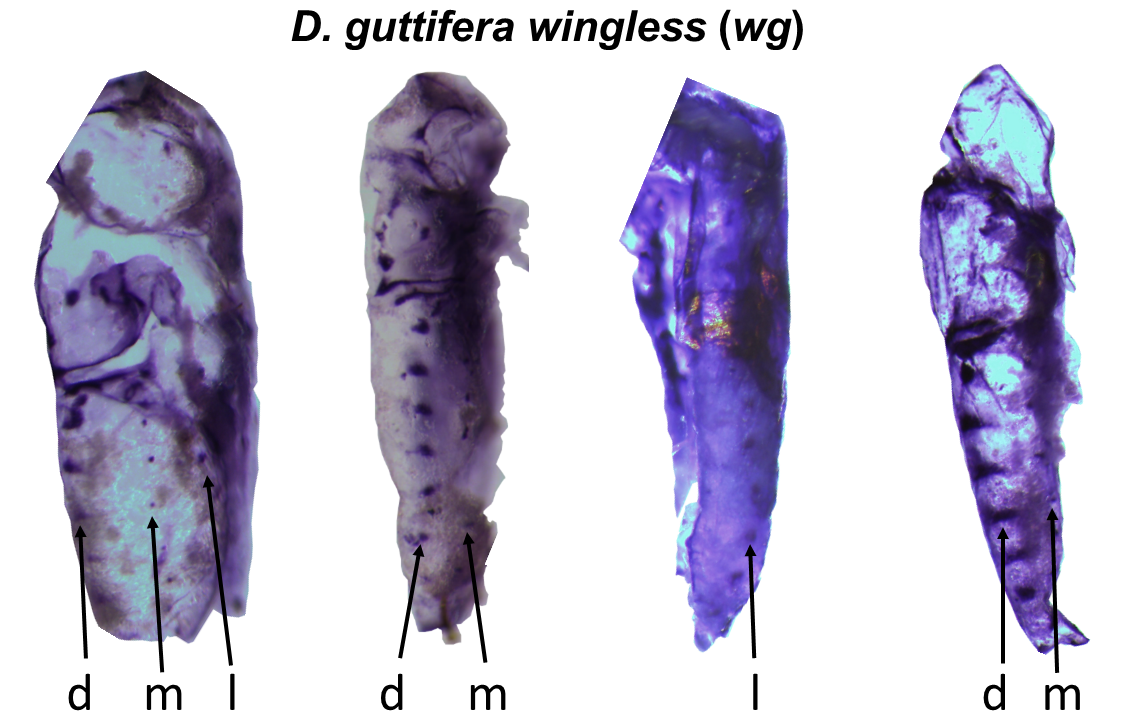

Supplement: S4 Fig — (TIF) [file pone.0279061.s004.tif]

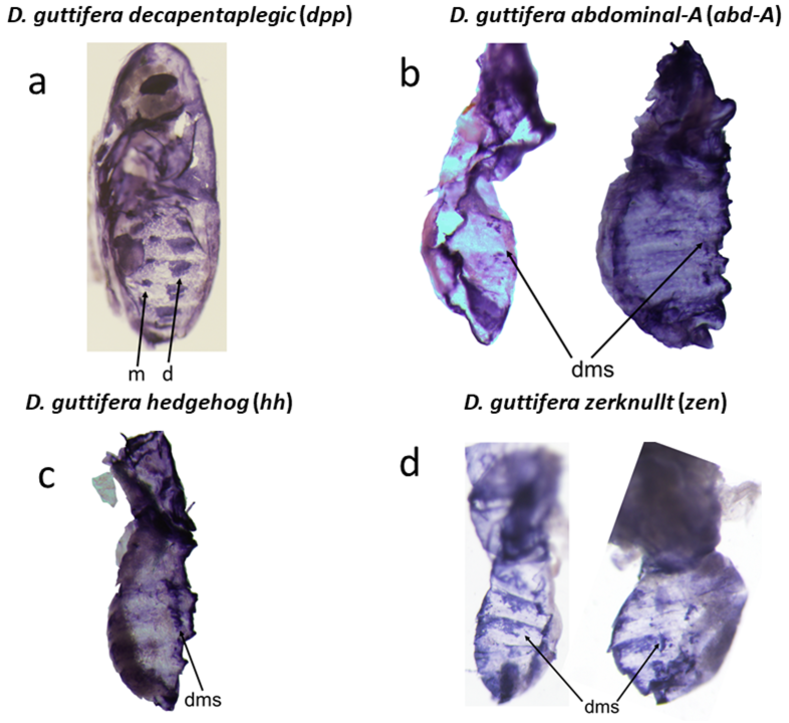

Supplement: S5 Fig — a, D. guttifera P8 pupa stained with a dpp probe. b, D. guttifera P9 pupa (left) and P10 pupa (right) stained with an abd-A probe. c, D. guttifera P10 pupa stained with a hh probe. d, Two different D. guttifera P10 pupae stained with a zen probe. dms = dorsal midline shade, d = dorsal, m = median row of spots. (TIF) [file pone.0279061.s005.tif]

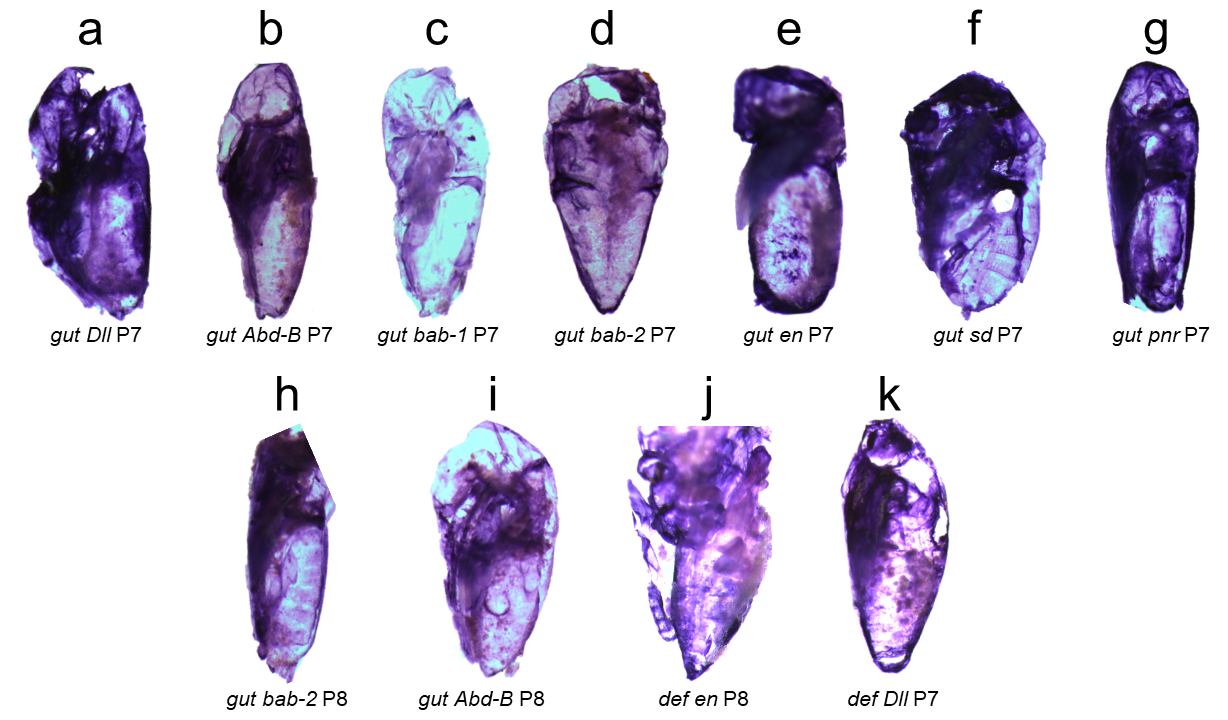

Supplement: S6 Fig — (TIF) [file pone.0279061.s006.tif]

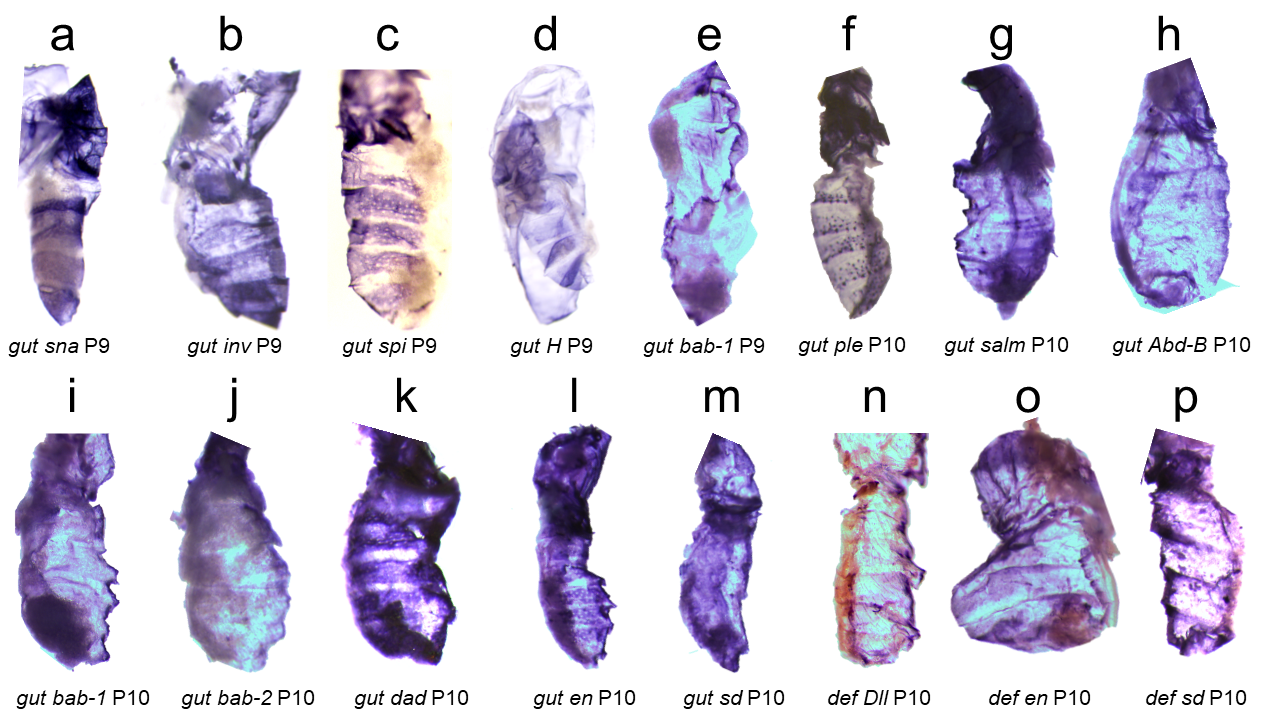

Supplement: S7 Fig — (TIF) [file pone.0279061.s007.tif]

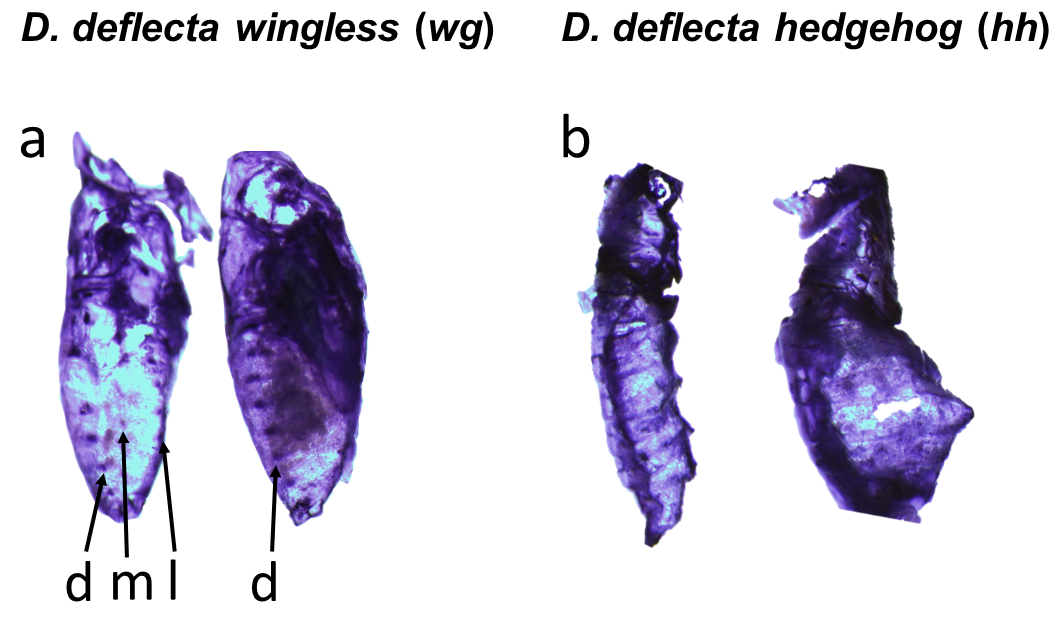

Supplement: S8 Fig — a, Two different D. deflecta P9 pupae stained with a wg probe. b, Two different D. deflecta P10 pupae stained with a hh probe. (TIF) [file pone.0279061.s008.tif]

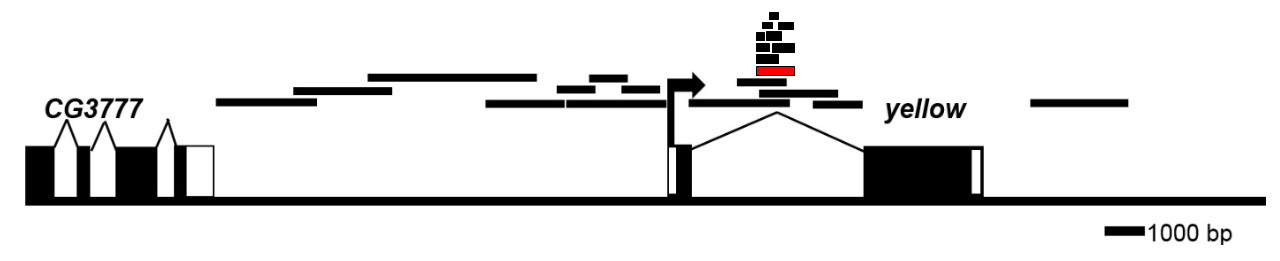

Supplement: S9 Fig — The horizontal bars indicate the DNA fragments of the D. guttifera y gene that were tested in transgenic D. guttifera for regulatory activity. Red: gut y spot CRM. (TIF) [file pone.0279061.s009.tif]

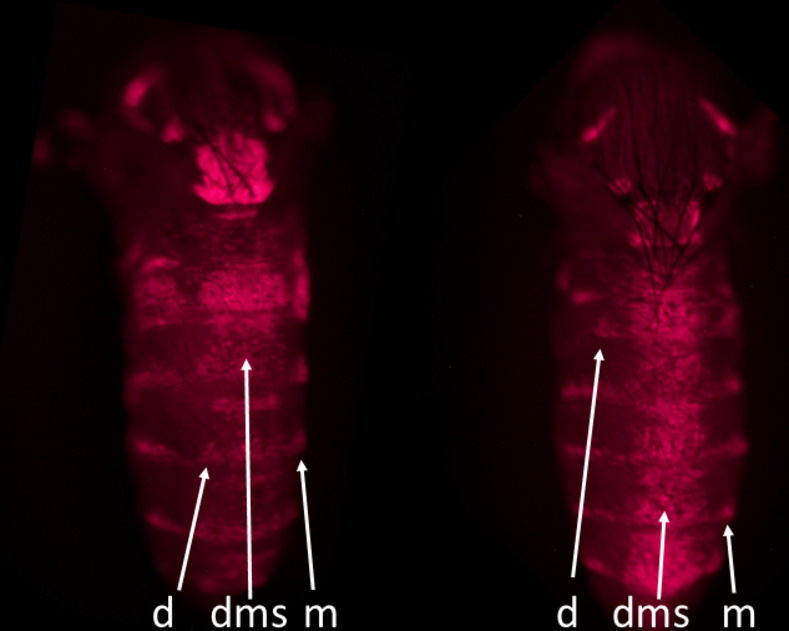

Supplement: S10 Fig — (TIF) [file pone.0279061.s010.tif]

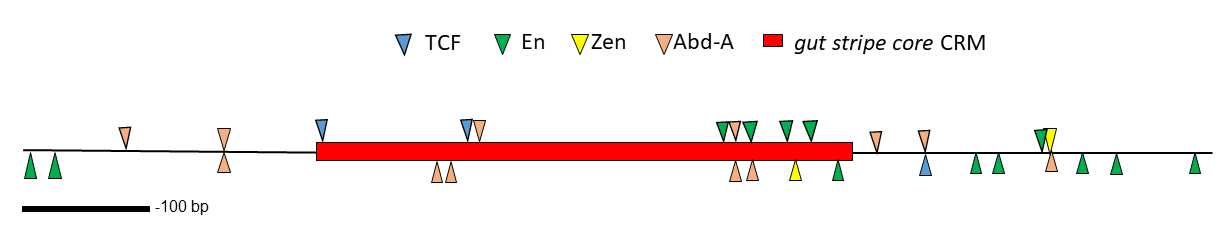

Supplement: S11 Fig — The red solid bar is the gut y core stripe CRM. (TIF) [file pone.0279061.s011.tif]

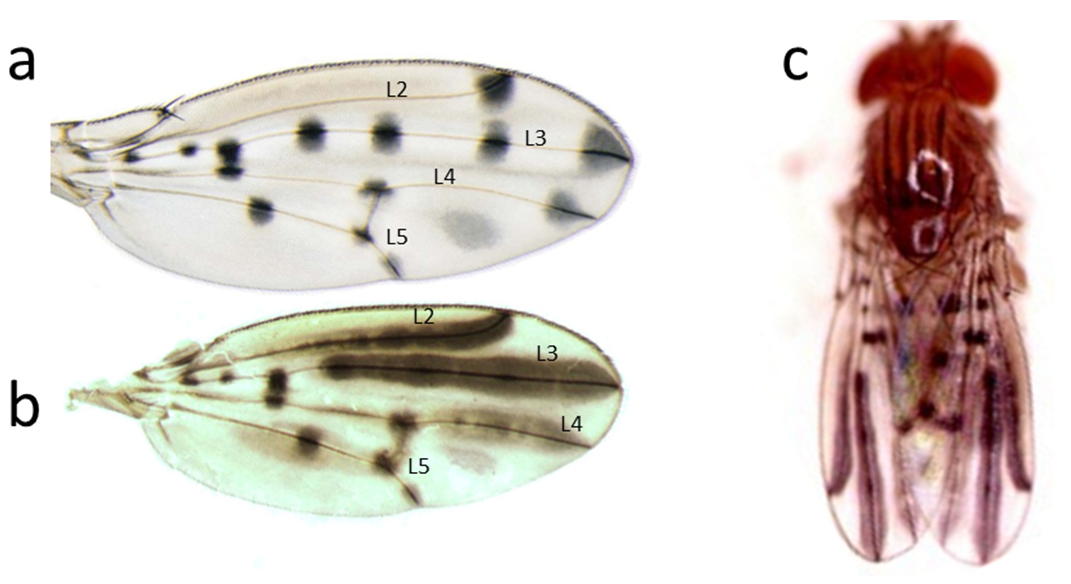

Supplement: S12 Fig — a, The wing of an adult D. guttifera, wild type. b, c, Ectopic wing pigmentation of adult D. guttifera expressing the wg cDNA construct. (TIF) [file pone.0279061.s012.tif]

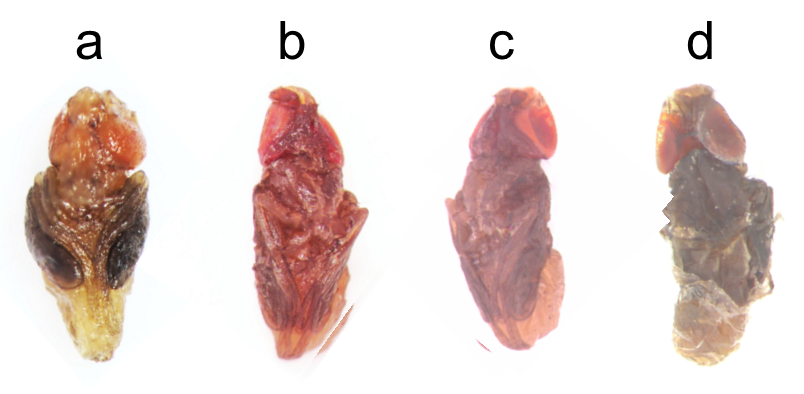

Supplement: S13 Fig — a, b, Knockdown of wg mRNA in D. guttifera at pupal stages P7 and P8. c, d, Knockdown of dpp mRNA in D. guttifera at pupal stage P8. (TIF) [file pone.0279061.s013.tif]

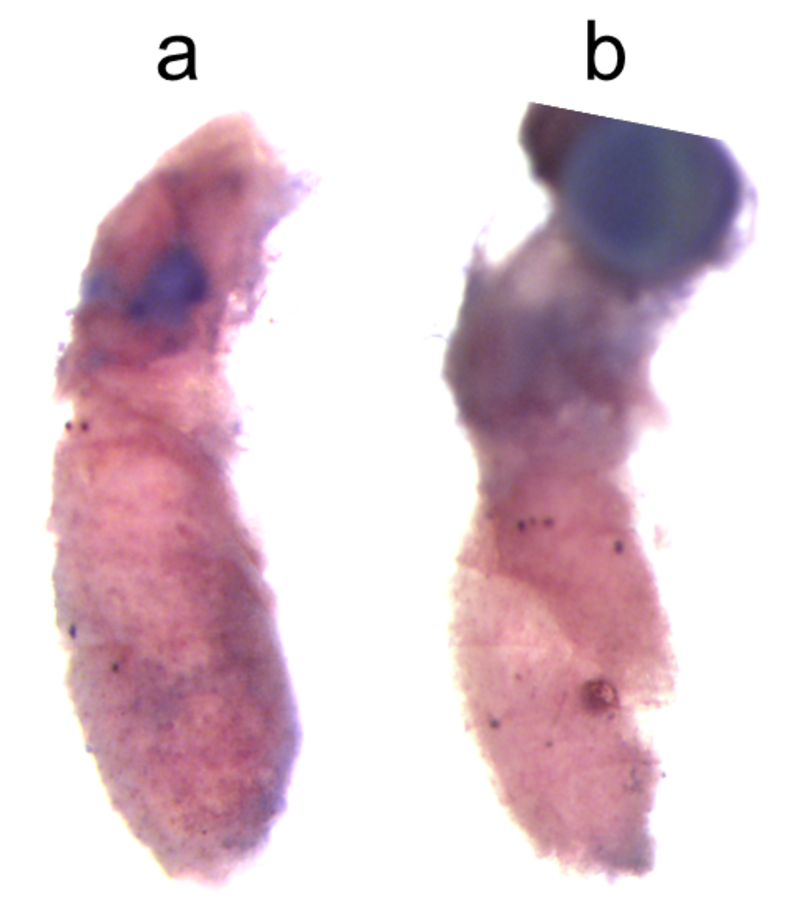

Supplement: S14 Fig — ISH showing the lack of ectopic expression of the D. melanogaster-derived wg (a) and hh (b) genes in wildtype (non-transgenic) D. guttifera pupae at stage P10, acting as the negative controls for the results shown in Fig 9. (TIF) [file pone.0279061.s014.tif]

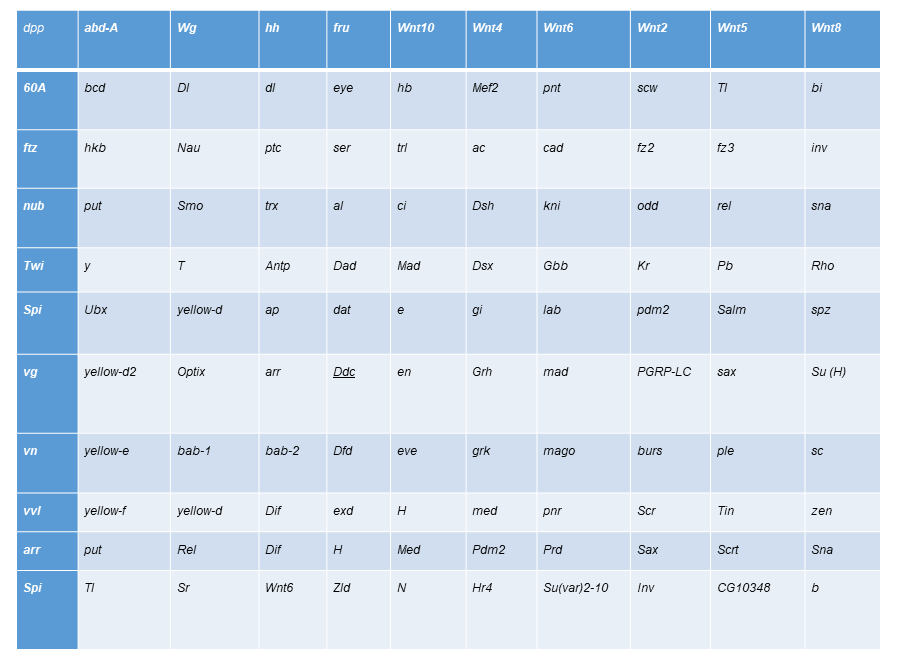

Supplement: S1 Table — (TIF) [file pone.0279061.s015.tif]

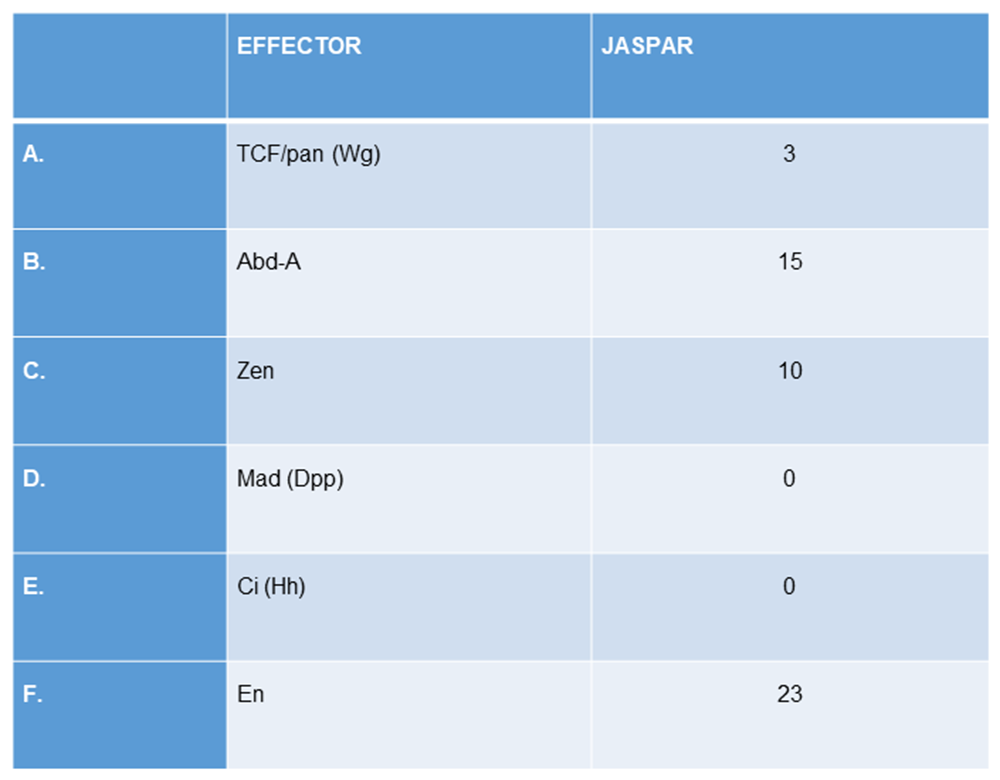

Supplement: S2 Table — This table was extrapolated from the data in S9 Fig. (TIF) [file pone.0279061.s016.tif]
