# Supplementary material for: The regulation of a pigmentation gene in the formation of complex color patterns in Drosophila abdomens
Source: PLoS One. 2022 Dec 19;17(12):e0279061. doi: 10.1371/journal.pone.0279061 (PMC9762589; doi:10.1371/journal.pone.0279061)
Supplement: S1 File — (PDF) [file pone.0279061.s017.pdf]

D. guttifera *D. guttifera*: 1-953 (+)

D. deflecta *D. deflecta*: 1-938 (+) The sequence identity between gut y spot CRM and def y spot CRM is 74%

```
000000001  AATTGTGAAGACAAAAAACCAGCAGCTGCGGTTGAGTACGACATTTATTGGCTATACAAT  000000060
>>>>>>>>>                     |||| ||||||||| |||| ||||  |||||||| || |||  <<<<<<<<<
000000001  -------------------CAGCTGCTGCGGTTCAGTA-GACAACTATTGGCTTTATAAT  000000040

000000061  GAGGTTGTCAAGTGGCCACAATTTTAATATCAA-----TCAATGTTGACCTCTCACTCTC  000000115
>>>>>>>>>  ||||||||||||||||||||||   ||||||||     |||||||| |||||||        <<<<<<<<<
000000041  GAGGTTGTCAAGTGGCCACAAT---AATATCAACACAATCAATGTTAACCTCTC------  000000091

000000116  CCAGTGTTCAA--AAAGACTCAAATGAATGAAT----ACTTGAATAT-----------AA  000000158
>>>>>>>>>        | |||  |||||||| ||||||| | |    ||||||||||           ||  <<<<<<<<<
000000092  ------TCCAATCAAAGACTCGAATGAATAATTGACCACTTGAATATGGTATGTGTACAA  000000145

000000159  CAAGATCATTATTAGT-TTTGTTGTGCGACAGTTTTGAATAACGCTCAAGTTCTCACATT  000000217
>>>>>>>>>  ||| || ||||||      | || |||||||||||||||||| |||||||||||||||||  <<<<<<<<<
000000146  CAATATTATTATTGTCGCCTATTATGCGACAGTTTTGAATAATGCTCAAGTTCTCACATT  000000205

000000218  ATTTTGAGAATCGATTTC-------------------TGTCATGTCAGTCG---------  000000249
>>>>>>>>>  |     |||||||| |||                   |||||||||| | |           <<<<<<<<<
000000206  ACCA-AAGAATCGAATTCGGAGACAGGGCAAATTGTGTGTCATGTCATTTGCAATTGGAT  000000264

000000250  ---AAATTGAAATTGAAAGAAAATCCATTGAATAAAAAATTAATATCGCTGTTTTATAAA  000000306
>>>>>>>>>     ||| |||||||||| ||| |||| |||||| | |||||| ||||  || |||| |||  <<<<<<<<<
000000265  TTGAAACTGAAATTGAACGAATATCCGTTGAATTACAAATTATTATCAATGATTTACAAA  000000324

000000307  TGAAGCTCAGTGAGCCGCTTTGGCTTTTATGTGCTTTATGTGATTTGATTTGAACAAGCT  000000366
>>>>>>>>>   |||||||| ||||| ||||  ||||||||| |||||||||| |||    |   |   ||  <<<<<<<<<
000000325  CGAAGCTCACTGAGCTGCTTAAGCTTTTATGCGCTTTATGTGTTTT----TTGCCGCACT  000000380

000000367  GAAAGCCAAAAAGCCAA------------TTGCTTTTAGTTAATCGCCAGTCAATAATGG  000000414
>>>>>>>>>  |||||||||||||  ||             ||||||||  |||| |||||||||||||||  <<<<<<<<<
000000381  GAAAGCCAAAAAGTTAAATGGCCAACTGGCTGCTTTTAACTAATAGCCAGTCAATAATGG  000000440

000000415  CAAAATTCATTGGTCGTTTGCAATTTGCAAATGCGTCAAGTGATGTTAATTTTTT-----  000000469
>>>>>>>>>   ||||||||||||||||||||||| ||||||||||||| |  |||| ||| || |       <<<<<<<<<
000000441  -AAAATTCATTGGTCGTTTGCAATGTGCAAATGCGTCAGGCAATGTAAATGTTGTCGCCT  000000499

000000470  -----------------------------TGCGGAACAGGTTCCGATCGGAACCACCAAA  000000500
>>>>>>>>>                               |||||||||||||| |||||||||  |||||  <<<<<<<<<
000000500  GCATTTGTTTCTTTGATTTTTCTCGAATGTGCGGAACAGGTTCAGATCGGAACAGCCAAA  000000559
```

```
000000501  AAACTGAATGAATGAATGCGATTTGTT---------------TGTCAAGTCAACAGGAAA  000000545
>>>>>>>>>  ||          |||||| |||| |||               | ||||||||||||||||  <<<<<<<<<
000000560  AA----------TGAATGAGATTCGTTAGGCAGCTGCGACAATATCAAGTCAACAGGAAA  000000609

000000546  CTTCAATCAAAAGCATCTGAAACTTAAACGCCGACTACTATTTCAATTTGCCACACTTTC  000000605
>>>>>>>>>  |||| ||||||  |||| |||||||||||  |||||||||||||||||||||||||||||  <<<<<<<<<
000000610  CTTCCATCAAATCCATCCGAAACTTAAAC-TCGACTACTATTTCAATTTGCCACACTTTC  000000668

000000606  TGTCGACTAAATTGCACAAATTAAAGCCATGCTCAGGCAATTGACAAGCTGTCCGAGCCG  000000665
>>>>>>>>>  |||| |||||||||||||||||||||| ||||||||||  |||||||||||||         <<<<<<<<<
000000669  TGTCTACTAAATTGCACAAATTAAAGCAATGCTCAGGCTTTTGACAAGCTGTC-------  000000721

000000666  AAATCAGATTCAGTTTCAGTCTCAGATTCAGATTCAGGATATCAGGTACAGGATGTAGGA  000000725
>>>>>>>>>                       ||||||  ||| |||||||||||||||||||||||||||  <<<<<<<<<
000000722  ---------------------TCAGAT--AGAGTCAGGATATCAGGTACAGGATGTAGGA  000000758

000000726  TGTTGTTTAACCCGACAGCTGGACAACTGATCGTCCAGTTAGCAGAGAGCGAAAGAGAGA  000000785
>>>>>>>>>  |||||||| ||||||||||||||||||||||||| |||||||  |  |    ||||||    <<<<<<<<<
000000759  TGTTGTTTCACCCGACAGCTGGACAACTGATCGTTCAGTTAGTGGGCA----AAGAGA--  000000812

000000786  GCAATTGAGCCATAACCCCAGTTGACAGCTTGATAGGTTGACAGGCCATCTAAAATATGG  000000845
>>>>>>>>>      |||||||||  ||||||||||||||||||        || ||||||||||        <<<<<<<<<
000000813  ----TTGAGCCATTGCCCCAGTTGACAGCTTGA--------CAAGCCATCTAAA------  000000854

000000846  TGCACAGTCCACTTAGCGTGGTGAAATTTCAGTTCATACGAATGTGATTGACACTGTCAC  000000905
>>>>>>>>>  | |||| |||||||||||||||||||||||   |  |||||||||||||||||||||||   <<<<<<<<<
000000855  TACACACTCCACTTAGCGTGGTGAAATTTCGAATT-TACGAATGTGATTGACACTGTCAT  000000913

000000906  GAAAGCAAAACACATGTGGACACAAGTCCGTCGGGTTAGACAATCTAA  000000953
>>>>>>>>>  |||||| | ||||| || |                        | |    <<<<<<<<<
000000914  GAAAGCTAGACACACGTTGG-----------------------TTTGC  000000938
```

annotation

ATGC Exon

ATGC UTR

conservation

ATGC CNS

ATGC Exon

ATGC UTR
