# Supplementary material for: The regulation of a pigmentation gene in the formation of complex color patterns in Drosophila abdomens
Source: PLoS One. 2022 Dec 19;17(12):e0279061. doi: 10.1371/journal.pone.0279061 (PMC9762589; doi:10.1371/journal.pone.0279061)
Supplement: S2 File — The gut y core stripe CRM is highlighted in red. (PDF) [file pone.0279061.s018.pdf]

52 putative sites were predicted with these settings (80%) in sequence named
***def y* spot CRM**

Model ID	Model name	Score	Relative score	Start	End	Strand	predicted site sequence
MA0220.1	en	6.041	0.827058165046636	32	38	-1	TTGATTG
MA0220.1	en	6.041	0.827058165046636	53	59	-1	TTGATTG
MA0256.1	zen	7.003	0.850509160267458	65	71	1	CGAATGA
MA0206.1	abd-A	5.810	0.810777134708393	72	78	1	ATAATTG
MA0220.1	en	8.445	0.915937118075064	72	78	1	ATAATTG
MA0206.1	abd-A	6.903	0.850089405954999	73	79	-1	TCAATTA
MA0220.1	en	6.384	0.839739313502855	73	79	-1	TCAATTA
MA0206.1	abd-A	5.723	0.807647978625635	109	115	1	ATTATTA
MA0256.1	zen	5.769	0.805380768019751	110	116	-1	ATAATAA
MA0237.2	pan	6.902	0.801861090541118	130	143	1	GCGACAGTTTTGAA
MA0237.2	pan	8.270	0.825699147885663	164	177	-1	TCGATTCTTTGGTA
MA0220.1	en	5.634	0.812010854779343	192	198	1	CAAATTG
MA0256.1	zen	7.003	0.850509160267458	208	214	-1	CAAATGA
MA0220.1	en	5.634	0.812010854779343	215	221	-1	CCAATTG
MA0206.1	abd-A	6.903	0.850089405954999	252	258	1	TGAATTA
MA0220.1	en	6.384	0.839739313502855	252	258	1	TGAATTA
MA0220.1	en	5.976	0.824655031957265	259	265	1	CAAATTA
MA0256.1	zen	6.003	0.813938339969478	259	265	1	CAAATTA
MA0206.1	abd-A	5.723	0.807647978625635	262	268	1	ATTATTA
MA0256.1	zen	5.769	0.805380768019751	263	269	-1	ATAATAA
MA0206.1	abd-A	6.016	0.818186400835384	269	275	1	TCAATGA
MA0256.1	zen	6.266	0.823556465707847	269	275	1	TCAATGA
MA0237.2	pan	7.957	0.820244972484462	278	291	-1	GAGCTTCGTTTGTA
MA0206.1	abd-A	5.548	0.801353699148823	353	359	1	TTAAATG
MA0220.1	en	6.041	0.827058165046636	353	359	1	TTAAATG
MA0206.1	abd-A	6.981	0.852894856236092	375	381	1	TTAACTA
MA0220.1	en	6.384	0.839739313502855	375	381	1	TTAACTA
MA0206.1	abd-A	6.903	0.850089405954999	376	382	-1	TTAGTTA
MA0220.1	en	6.384	0.839739313502855	376	382	-1	TTAGTTA

MA0220.1	en	5.758	0.816595293288297	379	385	1	CTAATAG
MA0206.1	abd-A	6.016	0.818186400835384	390	396	-1	TTATTGA
MA0256.1	zen	6.266	0.823556465707847	390	396	-1	TTATTGA
MA0220.1	en	5.650	0.812602395232112	393	399	1	ATAATGG
MA0220.1	en	6.041	0.827058165046636	404	410	1	TTCATTG
MA0256.1	zen	7.003	0.850509160267458	405	411	-1	CCAATGA
MA0535.1	Mad	6.111	0.809414886673957	445	459	-1	CAGGCGACAACATTT
MA0206.1	abd-A	6.016	0.818186400835384	521	527	1	TGAATGA
MA0256.1	zen	6.266	0.823556465707847	521	527	1	TGAATGA
MA0256.1	zen	7.003	0.850509160267458	531	537	-1	CTAACGA
MA0220.1	en	5.634	0.812010854779343	611	617	-1	CAAATTG
MA0220.1	en	6.041	0.827058165046636	635	641	1	TAAATTG
MA0220.1	en	5.976	0.824655031957265	644	650	1	CAAATTA
MA0256.1	zen	6.003	0.813938339969478	644	650	1	CAAATTA
MA0206.1	abd-A	7.150	0.858973331845128	645	651	-1	TTAATTT
MA0220.1	en	7.153	0.868170226514028	645	651	-1	TTAATTT
MA0220.1	en	5.634	0.812010854779343	753	759	-1	CTAACTG
MA0206.1	abd-A	6.048	0.819337354796858	809	815	1	CTAAATA
MA0220.1	en	5.976	0.824655031957265	809	815	1	CTAAATA
MA0256.1	zen	6.003	0.813938339969478	809	815	1	CTAAATA
MA0220.1	en	5.634	0.812010854779343	823	829	-1	CTAAGTG
MA0206.1	abd-A	6.176	0.823941170642755	869	875	-1	TTCATGA
MA0256.1	zen	6.266	0.823556465707847	869	875	-1	TTCATGA

Comment: This type of analysis has a high sensitivity but abysmal selectivity. In other words: while true functional will be detected in most cases, most predictions will correspond to sites bound in vitro but with no function in vivo. A number of additional contraints of the analysis can improve the prediction; phylogenetic footprinting is the most common. We recommend using the ConSite service, which uses the JASPAR datasets.

The review Nat Rev Genet. 2004 Apr;5(4):276-87 gives a comprehensive overview of transcription binding site prediction
