# Supplementary material for: The regulation of a pigmentation gene in the formation of complex color patterns in Drosophila abdomens
Source: PLoS One. 2022 Dec 19;17(12):e0279061. doi: 10.1371/journal.pone.0279061 (PMC9762589; doi:10.1371/journal.pone.0279061)
Supplement: S3 File — (DOCX) [file pone.0279061.s019.docx]

**PCR primer sequences**

We used the following primers to amplify the CRM sequences:

(i) *gut y* *spot* CRM: Fwd: 5’-CAGCTGCGGTTGAGTACGAC-3’and

Rvs: 5’-GCCAACTCGACGGGAATTC-3’. Restriction sites: KpnI and SacII.

(ii) *def y spot* CRM: Fwd: 5’-CAGCTGCTGCGGTTCAGTAG-3’ and

Rvs: 5’-GCTAGACACACGTTGGTTTGCT-3’. Restriction sites: KpnI and SacII.

(iii) *gut* *y spot* CRM sub-fragment #1: Fwd: 5’-CAGCTGCGGTTGAGTACGAC-3’ and

Rvs: 5’-ACTGAATCTGATTTCGGCTCG-3’. Restriction sites: KpnI and SacII.

(iv) *gut* *y spot* CRM sub-fragment #2: Fwd: 5’-AGTTAATCGCCAGTCAATAATGGC-3’ and

Rvs: 5’- GAATTCCCGTCGAGTTGGC-3’. Restriction sites: KpnI and SacII.

(v) *gut* *y* *spot* CRM sub-fragment #3: Fwd: 5’-CAGCTGCGGTTGAGTACGAC-3’ and

Rvs: 5’-GCCATTATTGACTGGCGATTAAC-3’. Restriction sites: KpnI and SacII.

(vi) *gut* *y* *spot* CRM sub-fragment #4: Fwd: 5’-AAATGAAGCTCAGTGAGCCGC-3’ and

Rvs: 5’-ACTGAATCTGATTTCGGCTCG-3’. Restriction sites: KpnI and SacII.

(vii) *gut* *y* *spot* CRM sub-fragment #5: Fwd: 5’-AGCATCTGAAACTTAAACGCCG-3’ and

Rvs: 5’-GAATTCCCGTCGAGTTGGC-3’. Restriction sites: KpnI and SacII.

(viii) *gut* *y* *spot* CRM sub-fragment #6: Fwd: 5’-CAGCTGCGGTTGAGTACGAC-3’ and

Rvs: 5’-CAGCGATATTAATTTTTTATTCAATGG-3’. Restriction sites: KpnI and SacII.

(ix) *gut y* *spot* CRM sub-fragment #7 (*gut y core stripe* CRM):

Fwd: 5’-AAATGAAGCTCAGTGAGCCGC-3’ and

Rvs: 5’-GCGATTTGTTTGTCAAGTCAAC-3’. Restriction sites: KpnI and SacII.

(x) *gut y* *spot* CRM sub-fragment #8: Fwd: 5’-AAATGAAGCTCAGTGAGCCGC-3’ and

Rvs: 5’-GTTGACTTGACAAACAAATCGC-3’. Restriction sites: KpnI and SacII.

(xi) *def y* *spot* CRM sub-fragment #1: Fwd: 5’-CAGCTGCTGCGGTTCAGTAG-3’ and

Rvs: 5’-ATTGTCGCAGCTGCCTAACG-3’. Restriction sites: KpnI and SacII.

(xii) *def y* *spot* CRM sub-fragment #2: Fwd: 5’-AACGAAGCTCACTGAGCTGC-3’ and

Rvs: 5’-AGCAAACCAACGTGTGTCTAGC-3’. Restriction sites: KpnI and SacII.

(xiii) *def y* *spot* CRM sub-fragment #3: Fwd: 5’-CAGCTGCTGCGGTTCAGTAG-3’ and

Rvs: 5’-GTTAAAAGCAGCCAGTTGGCC-3’. Restriction sites: KpnI and SacII.

(xiv) *def y* *spot* CRM sub-fragment #4: Fwd: 5’-CAAAGAATCGAATTCGGAGACAG-3’ and

Rvs: 5’-ATTGTCGCAGCTGCCTAACG-3’. Restriction sites: KpnI and SacII. (Clone name: *def y* 1.1C2)

(xv) *def y* *spot* CRM sub-fragment #5: Fwd: 5’-GAATGAGATTCGTTAGGCAGC-3’ and

Rvs: 5’-AGCAAACCAACGTGTGTCTAGC-3’. Restriction sites: KpnI and SacII.

(xvi) *def y* *spot* CRM sub-fragment #6: Fwd: 5’-CAGCTGCTGCGGTTCAGTAG-3’ and

Rvs: 5’-TTCAACGGATATTCGTTCAATTTC-3’. Restriction sites: KpnI and SacII.

(xvii) *def y* *spot* CRM sub-fragment #7 (*def y* *core stripe* CRM):

Fwd: 5’-CAAAGAATCGAATTCGGAGACAG-3’ and

Rvs: 5’-GTCAGGCAATGTAAATGTTGTCG-3’. Restriction sites: KpnI and SacII.

(xviii) *def y* *spot* CRM sub-fragment #8: Fwd: 5’-AACGAAGCTCACTGAGCTGC-3’ and

Rvs: 5’-ATTGTCGCAGCTGCCTAACG-3’. Restriction sites: KpnI and SacII.

These forward and reverse primer sequences do not include restriction sites.

We used the following primers to amplify the coding region of the genes to synthesize ISH probes:

*gut def y* ex2 Fwd: 5’-CCAACATCGCCGTGGACATTG-3’

*gut def y* ex2 Rvs: 5’-AATTGCGGAGTGTACGGCATCG-3’

*mel y* ex2 Fwd: 5’-CTAACATTGCCGTGGATATAGGC-3’

*mel y* ex2 Rvs: 5’-AATTGCGGTGAGTACGGCATTG-3’

*gut hh* ex3 Fwd: 5’-GTGAGCAGTGTTCAGAGTCG-3’

*gut def hh* ex3 Rvs: 5’-TACATATTGTATAGGGTATCTGTCTG-3’

*def hh* ex3 Fwd: 5’-GTAAGCAGTGTCCAAAGACGC-3’

*mel wg* ex4 Fwd: 5’-CACGTCCAAGCGGAGATGCG-3’

*mel wg* ex4 Rvs: 5’-GGCGACGGCATGTTCGGGTG-3’

*gut def abd-A* ex1 Fwd: 5’-TTCGCTGCTCGTAACGCCACAAC-3’

*gut abd-A* ex1 Rvs: 5’-TTTGCTGTCGACGCTGGCAGCG-3’

*def abd-A* ex1 Rvs: 5’-TTTGCTGTCGACGTTGGCAGCG-3’

*gut def dpp* ex3 Fwd: 5’-CATCGGGAGCCGAGCTATTGGC-3’

*gut def dpp* ex3 Rvs: 5’-GCATCATAGCCCGCTGGCGC-3’

*gut def zen* ex2 Fwd: 5’-CAGTTGGTGGAACTGGAGCAGG-3’

*gut zen* ex1 Rvs: 5’-TGCTACTGGACACTGCCACTGG-3’

**RNA *in situ* hybridization**

The gene expression patterns of candidate genes in developing *D*. *guttifera* pupae (stages P6 to P15) were investigated using RNA *in situ* hybridization. Anti-sense RNA probes were made by amplifying partial protein-coding sequences using PCR from genomic DNA. These PCR products were cloned into pGEM-TEasy vector. The Digoxigenin-labeled RNA probes were made by re-amplifying the PCR products followed by *in vitro* transcription. The pupal abdomens were incubated with the probe (1:500) and subjected to hybridization at 65ºC for at least 18 h. The abdomens were later stained using NBT/BCIP solutions (Promega). The gene expression patterns were imaged, using an Olympus SZX16 stereo microscope and a digital camera. We used 30 to 40 pupal halves for each *in situ* hybridization experiment. We performed three independent repeats for the 110 genes that we showed in S1 Table. The genes that showed a positive correlation with the pigment pattern were further tested 3 more times. Only genes that showed a consistent pattern in all our three repeats are included in this manuscript.
