# Supplementary material for: The regulation of a pigmentation gene in the formation of complex color patterns in Drosophila abdomens
Source: PLoS One. 2022 Dec 19;17(12):e0279061. doi: 10.1371/journal.pone.0279061 (PMC9762589; doi:10.1371/journal.pone.0279061)
Supplement: Attachment — Submitted filename: Response to reviewers.docx [file pone.0279061.s020.docx]

We sincerely thank the reviewers for their valuable comments and their efforts to improve this manuscript. Our responses to the new comments by the reviewers are given below:

**Reviewer #1**

Reviewer #1: The added images and increased information on CRM dissection have resolved my main concerns. A few small suggestions:

**Comment:** Figure 5: I understand the difficulty of these dissections and stainings and have kept that in mind when evaluating your evidence. It's a shame you can't provide a better stain for dpp in 5c, but it at least has a few clearer spots towards the anterior and posterior of the thorax. I think adjusting the arrow towards a more convincing spot would improve interpretability for this part of the story.

**Response:** We have adjusted the arrow pointing at the median row in Figure 5; it is now pointing at more convincing spot.

**Comment:** Figure 6: Panel e looks much better. However it looks like orientation of b and c were flipped compared to the initial submission. I believe they were "correct" (dorsal left) before.

**Response:** We thank the reviewer for this comment. We have changed the orientation of Figure 6 b and c so that they match the orientation of Figure 6 e.

**Comment:** Figure 7: In panel b shouldn't fragment 7 also be shaded pink?

**Response:** Yes, we agree that Fragment 7 in Figure 7 should be shaded, and we have shaded the bar.

**Reviewer #2**

Reviewer #2: The revised manuscript by Raja and colleagues “The regulation of a pigmentation gene in the formation of complex color patterns in Drosophila abdomens” describes the analyses of abdomens pigmentation in D. guttifera with an extension to a few other species in the quinaria species group. They focus on the yellow (y) gene since it shows a high correlation with abdomen pigmentation. The authors found five genes (wg, dpp, abd-A, hh, and zen) that show different degrees of spatiotemporal correlation between the expression of these genes and pigmentation patterns. They identified an intronic enhancer from D. guttifera that recapitulates the y patterning. They also showed the evolutionary conservation of the orthologous enhancer from D. deflecta for the core stripes. The authors tried functional analyses by expressing cDNAs of wg, hh, and abd-A. They also tried to target wg, hh, and dpp by RNAi. The functional studies did not provide the desired changes.

**Comment:** Unfortunately, the authors could not address the functional analyses of the regulatory network, mainly due to experimental difficulties associated with working in this challenging experimental system. At the same time, the authors write in the discussion “…the Jaspar binding analysis suggests that the developmental genes wg, dpp, zen, and abd-A are directly activate the gut y spot CRM.” In this case, the lab developed a protocol to generate transgenic D. guttifera flies (as shown here and in a previous publication), why not to generate transgenic reporter lines that lack the predicted biding sites? Or focus on some of these genes? This would have given some context to the proposed regulatory network of y. It may have also aided to address the evolutionary changes of the CRM in other flies.

**Response:** We thank the reviewer for this suggestion. However, the students who worked on this project have graduated and the lab is currently having shortage of funding to continue with this research. We use the word “may” in our discussion about upstream factors regulating *y* gene, inferring that we are not proposing any regulatory relationships. We also added a sentence “We acknowledge that we do not provide any functional evidence and that the regulatory relationships between the identified toolkit genes and the *y* gene are correlative” at the end of second paragraph in the Discussion section

**Reviewer #3**

Reviewer #3: The original manuscript by Raja et al. required major revisions to be suitable for publication in PLoS Genetics. Two independent reviewers (Reviewer 2 and myself) had very similar concerns and suggestions for improving the manuscript. Unfortunately, the revised manuscript does not address any of these concerns. I understand the challenges in obtaining the data that is required to prove the regulatory relationships between yellow and the co-expressed toolkit genes. However, this cannot serve as an excuse for (almost) completely ignoring reviewer’s comments and suggestions. Even the minor comments were not addressed properly, and the authors did not change the main conclusion of the manuscript despite the lack of experimental support. Therefore, I recommend rejecting this manuscript.

**Response:** To the best of our knowledge, we responded to all reviewers’ comments and concerns, both major and minor comments. We agreed with your concerns about the lack of functional data and explained why some of the suggestions could not be actualized.
